# Effect of THBS1 on the Biological Function of Hypertrophic Scar Fibroblasts

**DOI:** 10.1155/2020/8605407

**Published:** 2020-12-09

**Authors:** D. Jiang, B. Guo, F. Lin, Q. Hui, K. Tao

**Affiliations:** ^1^Reconstructive and Plastic Surgery, General Hospital of Northern Theater Command, Shenyang, China; ^2^Graduate School, Jinzhou Medical University, 40 Songpo Road, Jinzhou 121001, China

## Abstract

Hypertrophic scarring is a skin collagen disease that can occur following skin damage and is unlikely to heal or subside naturally. Since surgical treatment often worsens scarring, it is important to investigate the pathogenesis and prevention of hypertrophic scarring. Thrombospondin-1 (THBS1) is a matrix glycoprotein that can affect fibrosis by activating TGF-*β*1, which plays a key role in wound repair and tissue regeneration; therefore, we investigated the effects of THBS1 on the biological function of hypertrophic scar fibroblasts. THBS1 expression was measured in hypertrophic scars and adjacent tissues as well as normal fibroblasts, normal scar fibroblasts, and hypertrophic scar fibroblasts. In addition, THBS1 was overexpressed or silenced in hypertrophic scar fibroblasts to determine the effects of THBS1 on cell proliferation, apoptosis, and migration, as well as TGF-*β*1 expression. Finally, the role of THBS1 in hypertrophic scarring was confirmed *in vivo* using a mouse model. We found that THBS1 expression was increased in hypertrophic scar tissues and fibroblasts and promoted the growth and migration of hypertrophic scar fibroblasts as well as TGF-*β*1 expression. Interestingly, we found that si-THBS1 inhibited the occurrence and development of bleomycin-induced hypertrophic scars *in vivo* and downregulated TGF-*β*1 expression. Together, our findings suggest that THBS1 is abnormally expressed in hypertrophic scars and can induce the growth of hypertrophic scar fibroblasts by regulating TGF-*β*1. Consequently, THBS1 could be an ideal target for treating hypertrophic scarring.

## 1. Introduction

Hypertrophic scarring is part of the routine pathological process of wound healing but is also a type of skin collagen disease that manifests as abnormal extracellular matrix (ECM) accumulation and excessive fibroblast proliferation [1]. Hypertrophic scarring can be caused by skin trauma, surgery, inflammation, scalds, foreign bodies, and acne [2] and involves clinical features such as redness, pruritus, and varying degrees of pain. Unfortunately, these scars are unlikely to self-heal and do not subside naturally [3], while surgical treatment frequently leads to recurrence, further dysfunction, and a worsened appearance [4, 5]. Therefore, the pathogenesis and prevention of hypertrophic scars have become an important area of research in the fields of dermatology and plastic surgery.

Hypertrophic scarring is a type of fibrotic disease [5] whose occurrence and development are closely associated with numerous fibrosis-related genes [6]. Previous studies have revealed a correlation between scar tissue formation and collagen metabolism imbalance and found that hypertrophic scar fibroblasts have a greater ability to proliferate and synthesize ECM than normal fibroblasts. A variety of cell growth factors are known to regulate collagen formation and catabolism; in particular, transforming growth factor-*β*1 (TGF-*β*1) has been shown to enhance the formation of hypertrophic scars [7].

THBS1 (thrombospondin-1) is a 450 kDa single subunit homologous matrix glycoprotein that is composed of N- and C-terminal globular domains linked by a procollagen homologous region, a type I prolamin repeat sequence, a type II epidermal growth factor repeat sequence, and a type III calcium binding region repeat sequence. THBS1 was first discovered in platelet *α* granules but was since found to be produced by many other cell types and affect fibrosis by activating TGF-*β*1 [8]. Therefore, THBS1 is widely studied in renal, myocardial, and liver fibrosis diseases [9] and it has been found to play a positive role in various ophthalmic diseases, including xerophthalmia, ocular allergy, angiogenesis, lymphangiogenesis, wound healing, corneal transplantation, and infectious keratitis [10]. In particular, THBS1 has been shown to inhibit neovascularization in a variety of ways, including antagonizing VEGF, inducing vascular endothelial cell apoptosis, and regulating endothelial cell proliferation and migration [10, 11]. Importantly, THBS1 was shown to upregulate the activity of TGF-*β*1, which plays a key role in wound repair and tissue regeneration [10, 12, 13]; however, few studies have investigated THBS1 expression in hypertrophic scars.

In this study, we investigated the effects of THBS1 on the biological function of hypertrophic scar fibroblasts *in vitro* and confirmed the role of THBS1 in hypertrophic scarring and TGF-*β*1 *in vivo* using a mouse model. Abnormal THBS1 expression in hypertrophic scar tissue and fibroblasts was found to influence cell growth and migration, while THBS1 downregulation was able to inhibit the occurrence of hypertrophic scars.

## 2. Materials and Methods

### 2.1. Patients and Tissue Samples

The study was approved by the Medical Ethics Committee of the Northern Theater General Hospital. Between April 2016 and April 2018, 30 patients with hypertrophic scarring underwent surgical procedures at the Reconstructive and Plastic Surgery Department of the General Hospital of Northern Theater Command. The patients (25–58 years old) had a disease duration ranging from 6 to 24 months and included ten cases of hydrothermal scald, nine cases of flame burn, two cases of electric injury, and nine cases of cutting injury. The cases involved various body parts, namely, the upper limbs (*n* = 5), lower limbs (*n* = 5), chest (*n* = 10), and abdomen (*n* = 10). Hypertrophic scar (HS) and adjacent normal skin (normal) tissue specimens were collected for further experiments, while three additional normal scar tissue samples were obtained and collected during surgical procedures, all of which were common scars from trauma located in the upper limbs..

All samples were cleaned, and the epidermis and subcutaneous tissues were removed under aseptic conditions before the samples were divided into three parts. Total RNA was extracted from the first using RNAstore solution (Beyotime Biotechnology, Shanghai, China), while total protein was extracted from the second using protein lysate (Beyotime Biotechnology). The third was used to isolate and culture fibroblasts *in vitro*.

All procedures involving human participants were performed in accordance with the ethical standards of the institutional and/or national research committee as well as the 1964 Helsinki declaration and its later amendments or comparable ethical standards. Informed consent was obtained from all study participants.

### 2.2. Cell Culture

Under aseptic conditions, trimmed tissue was washed repeatedly, cut into fragments, and digested with 0.25% trypsin solution (Beyotime Biotechnology) at 4°C for approximately 8 h. The tissue was then inoculated into a clean culture dish with 4 mL of high-sugar medium (10% FBS; Thermo Fisher Scientific, Shanghai, China) and placed inside a constant-temperature incubator for 7 to 15 days until the cells began to adhere to the wall.

### 2.3. Western Blot

Tissue and cell samples were lysed with protein lysate, centrifuged at 12 000 rpm at 4°C for 20 min, and the protein concentration of the supernatant was determined. Lysates (50 *μ*g) were resolved using SDS-PAGE gels and transferred to nitrocellulose membranes by electroblotting. After incubation with 5% blocking solution for 1 h, the required bands were cut according to markers and incubated with antibodies against THBS1 (1 : 1000, Cell Signaling Technology, Cat no. 37879), TGF-*β*1 (1 : 1000, Abcam, ab92486), and GAPDH (1 : 1000, Cell Signaling Technology, Cat no. 60004) overnight at 4°C. After incubation with secondary antibodies (1 : 5000) at room temperature for 1 h, immunoreactivity was measured using a Western Lighting Ultra instrument (ECL, Pierce Technology, Shanghai, China).

### 2.4. RNA Extraction

Tissue or cell samples were disrupted for 5-20 s (2-3 times) before being mixed with RnaExTM reagent (0.7 mL) and split at room temperature for 5 min. The samples were then mixed with chloroform (0.2 mL), vortexed, and allowed to stand for 2 min at room temperature. After centrifugation at 12 000 rpm and 4°C for 10 min, the samples were mixed with anhydrous ethanol (200 *μ*L), centrifuged at 8000 rpm and 4°C for 1 min, mixed with buffer RWA (500 *μ*L), and centrifuged at 12 000 rpm and 4°C for 1 min. The samples were then air-dried for 2 min and incubated with DEPC water (50 *μ*L) at room temperature for 2 min, and RNA was collected by centrifugation at 12 000 rpm and 4°C for 1 min.

### 2.5. Reverse Transcription

RNA (1 mg) was reverse transcribed into cDNA in a 20 *μ*L system using a real-time reaction kit (Promega, Beijing, China). Real-time PCR was performed using an Mx3000P real-time PCR system (Applied Biosystems, Beijing, China).

### 2.6. Real-Time PCR

PCR was performed as follows: 40 cycles of 94°C for 15 s, 60°C for 10 s, and 72°C for 20 s. All procedures were repeated three times. The following forward (F) and reverse (R) primer sequences were used: THBS1, F: 5′-GTC ATA CAA CAC TCC CAC GC-3′; R: 5′-CCA GGG CAT AGG TAG AAG CT-3′; TGF-*β*1, F: 5′-ATG CCG CCC TCC GGG C-3′; R: 5′-TCA GCT GCA CTT GCA GGA GCG-3′; and GAPDH, F: 5′-AGC CAC ATC GCT CAG ACA C-3′; R: 5′-GCC CAA TAC GAC CAA ATC C-3′.

### 2.7. MTT Assays

Cells were seeded in 96-well plates at a density of 1 × 10^4^ cells/well and divided into two groups: the first was transfected with a plasmid or THBS1, while the second was transfected with si-NC and si-THBS1. After 0, 12, 24, 36, and 48 h, 10 *μ*L of MTT solution (5 mg/mL) was added and the medium was replaced with 150 *μ*L of dimethyl sulfoxide solution after incubation for 4 h at 37°C. Optical density was measured at 490 nm using a microplate reader (Bio-Rad Laboratories, Shanghai, China) [14] to determine cell proliferation.

### 2.8. Metastasis Assay

Cells were divided into two groups: the first was transfected with a plasmid or THBS1, while the second was transfected with si-NC and si-THBS1. A total of 1 × 10^5^ cells in 0.2 mL of serum-free DMEM were plated in the upper chamber of each plate (Merck, Hong Kong, China), whereas the lower chamber was filled with 0.6 mL of medium supplemented with 10% FBS. After incubation at 37°C for 24 h, the cells in the lower chamber were stained and counted under a high-power microscope.

### 2.9. Cell Apoptosis Analysis

To analyse cellular apoptosis, 2 × 10^5^ cells were seeded in plates and allowed to attach overnight. After treatment with annexin V-PI, apoptotic cells in different groups were detected using a FACSCalibur instrument (BD Biosciences, San Jose, CA, USA).

### 2.10. THBS1 Overexpression

THBS1 cDNA was cloned into multiple cloning sites of the pcDNA3.1 vector (Invitrogen, Carlsbad, CA, USA), and cells were transfected with the resultant expression vector and empty vector to establish THBS1 overexpression and control cell lines, respectively. The THBS1 plasmid was constructed with the following primer sequences: F, 5′-AGG GCA GGA AGA CTA TGA CAA G-3′; R, 5′-GCT GGG TTG TAA TGG AAT GG-3′.

### 2.11. sh-RNA Transfection

To silence THBS1, siTHBS1 (TAT CAT CTG GTA TAC CAT TGC, Shanghai GeneChem Company) and si-NC (control) were synthesized by GenePharma (Shanghai, China).

### 2.12. Mouse Model

Six-week-old BALB/c male mice (*n* = 12) received daily dorsal subcutaneous injections with bleomycin (100 *μ*g/100 *μ*L, dissolved in PBS) for two weeks, followed by daily subcutaneous injection with either si-NC or si-THBS1. After three weeks, the mice were sacrificed and their skin tissues were processed for analysis [15].

### 2.13. Haematoxylin and Eosin (H&E) Staining

After being fixed onto 10% formaldehyde pathological slides, samples were deparaffinized in xylene, rehydrated in decreasing ethanol concentrations, and stained with H&E as described previously [16]. Images were captured using a Nikon Eclipse E600 microscope (Nikon Instruments, Melville, NY, USA) with a 10x objective.

### 2.14. Immunofluorescence Staining

After being fixed onto 10% formaldehyde pathological slides, samples were deparaffinized in xylene, rehydrated in decreasing ethanol concentrations, and treated as described previously [17]. Briefly, the slides were blocked with 0.5% BSA for 30 min, incubated with THBS1 antibodies (1 : 200) overnight at 4°C, and then incubated with secondary antibodies (Vector Laboratories, Burlingame, CA, USA) for 1 h at room temperature. After treatment with avidin-biotin complex (Vector Laboratories) for 30 min at room temperature, the slides were counterstained with haematoxylin and imaged using a Nikon Eclipse E600 microscope (Nikon Instruments) with a 10x objective.

### 2.15. Statistical Analysis

All experiments were repeated three times. Data represent the mean ± standard error of the mean. Statistical analyses were carried out in GraphPad Prism 7 using Student's *t*-tests or one-/two-way ANOVA with Bonferroni's multiple comparison post hoc test. *P* values of <0.05 were considered to be statistically significant.

## 3. Results

### 3.1. THBS1 Expression in Hypertrophic Scar Tissues and Cells

**Figure 1:**
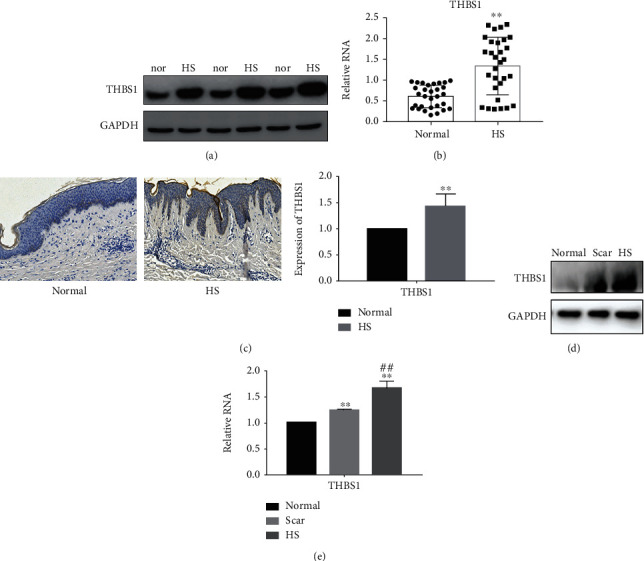
THBS1 expression in hypertrophic scar tissues and cells. (a, b) THBS1 expression in 30 hypertrophic scar (HS) and normal (nor) tissue samples was detected, respectively, by western blot and real-time PCR analyses. ^∗∗^*P* < 0.05. (c) THBS1 expression in 30 HS and normal tissue samples was detected, respectively, by immunohistochemical staining. ^∗∗^*P* < 0.05. Original magnification ×100. (d, e) THBS1 expression in normal, scar, and HS fibroblasts was detected by western blot and real-time PCR analyses. ^∗∗^*P* < 0.05 vs. normal fibroblasts.

Western blotting and real-time PCR revealed that THBS1 protein and mRNA levels were higher in hypertrophic scars than in normal tissues (Figures 1(a) and 1(b)), while immunohistochemical staining confirmed that THBS1 expression was higher in hypertrophic scars (Figure 1(c)). In addition, the analysis of normal, scar, and hypertrophic scar fibroblasts revealed that hypertrophic scar fibroblasts displayed the highest THBS1 expression (Figures 1(d) and 1(e)). We found that THBS1 was significantly up-regulated in hypertrophic scar tissue.

### 3.2. THBS1 Induced Hypertrophic Scar Cell Growth and Migration

**Figure 2:**
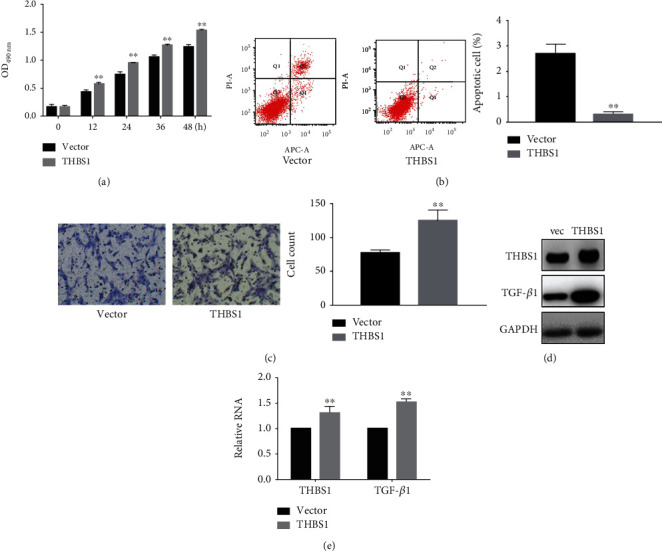
THBS1 promotes the growth and migration of hypertrophic scar cells. (a) Cell proliferation was assessed using MTT assays in hypertrophic scar cells 24 h after transfection with a vector or THBS1. ^∗∗^*P* < 0.05. (b) Apoptosis was detected using annexin V-PI assays in hypertrophic scar cells 24 h after transfection with a vector or THBS1. ^∗∗^*P* < 0.05. (c) Cell migration was determined using Transwell assays in hypertrophic scar cells 24 h after transfection with a vector or THBS1. ^∗∗^*P* < 0.05. Original magnification ×200. (d, e) THBS1 and TGF-*β*1 expression in hypertrophic scar cells transfected with a vector (vec) or THBS1. ^∗∗^*P* < 0.05.

To determine the effects of THBS1 on the biological function of hypertrophic scar cells, we constructed a plasmid-transfected fibroblast line overexpressing THBS1. MTT and apoptosis assays revealed that THBS1 significantly promoted cell proliferation (Figure 2(a)) and inhibited apoptosis (Figure 2(b)), respectively, while Transwell assays indicated that THBS1 stimulated migration (Figure 2(c)). Since TGF-*β*1 is a key protein downstream of THBS1, we examined THBS1 and TGF-*β*1 expression using western blotting and real-time PCR. Interestingly, transfection with THBS1 increased THBS1 and TGF-*β*1 expression (Figures 2(d) and 2(e)). Together, these results suggest that THBS1 promotes hypertrophic scar cell growth and migration. These results indicate that THBS1 can promote the proliferation and migration of fibroblasts.

### 3.3. si-THBS1 Inhibited Hypertrophic Scar Cell Growth and Migration

**Figure 3:**
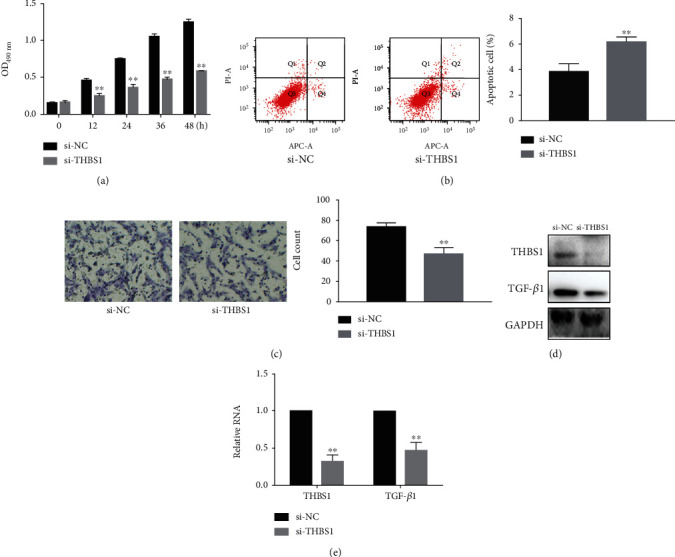
si-THBS1 inhibited hypertrophic scar cell growth and migration. (a) Proliferation was detected using MTT assays in hypertrophic scar cells 24 h after transfection with si-NC or si-THBS1. ^∗∗^*P* < 0.05. (b) Apoptosis was assessed using annexin V-PI assays in hypertrophic scar cells 24 h after transfection with si-NC or si-THBS1. ^∗∗^*P* < 0.05. (c) Migration was detected using Transwell assays in hypertrophic scar cells 24 h after transfection with si-NC or si-THBS1. ^∗∗^*P* < 0.05. Original magnification ×200. (d, e) THBS1 and TGF-*β*1 expression in hypertrophic scar cells transfected with si-NC or si-THBS1. ^∗∗^*P* < 0.05.

Next, we examined the effects of si-THBS1 on the proliferation, apoptosis, and migration of the THBS1-silenced hypertrophic scar fibroblasts. We observed significant differences in cell proliferation, apoptosis, and migration between the si-NC and si-THBS1 groups (Figures 3(a)–3(c)). In particular, si-THBS1 inhibited hypertrophic scar cell growth and migration, while western blotting and real-time PCR confirmed that si-THBS1 inhibited THBS1 and TGF-*β*1 expression (Figures 3(d) and 3(e)). These results indicate that si-THBS1 can inhibit the proliferation and migration of fibroblasts.

### 3.4. si-THBS1 Can Inhibit the Development of Hypertrophic Scars

**Figure 4:**
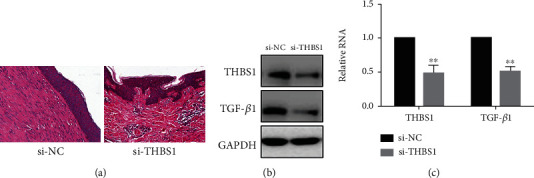
si-THBS1 can inhibit hypertrophic scar development: (a) HE staining images showing the dermal thickness of mouse skin treated with bleomycin with or without si-THBS1. Original magnification ×100; (b, c) THBS1 and TGF-*β*1 expression in hypertrophic scar tissues treated with si-NC and si-THBS1. ^∗∗^*P* < 0.05.

To investigate the role of THBS1 *in vivo*, we established a mouse model injected with si-THBS1 daily for three weeks in the bleomycin injection zone. H&E staining revealed that si-THBS1 significantly suppressed fibrosis (Figure 4(a)), while western blotting and real-time PCR indicated that si-THBS1 inhibited THBS1 and TGF-*β*1 expression *in vivo* (Figures 4(b) and 4(c)). These data show that si-THBS1 can inhibit the formation of hypertrophic scar in vivo.

## 4. Discussion

Hypertrophic scars are mainly treated using surgery, compression, radiation, cryopreservation, lasers, silica gel, hormone injection, or external drug application [18]; however, these methods are not very effective and can involve long treatment cycles, recurrence, serious complications, and induced tumours [19]. It is therefore important to identify an effective method for preventing and treating cicatricial hyperplasia. To elucidate the pathological process underlying hypertrophic scars, we conducted a series of experiments and propose that THBS1 may play a role in the development of hyperplastic scars.

Hypertrophic scars involve many types of prefibrotic cytokines, particularly the important regulatory factor TGF-*β*1 which was found to be regulated by the matricellular protein THBS1 [20, 21]. Although THBS1 is mainly released by activated platelets, it is also produced by other cells, such as fibroblasts, endothelial cells, and macrophages, during inflammation or stress [22]. In particular, studies have shown that patients with brachiocervical inflammatory myopathy associated with SS display increased serum THBS1 levels, confirming the correlation between THBS1 serum levels, macrophage infiltration, fibrotic replacement, and a reduced number of vessels in muscle biopsies [22]. It has also been shown that renal tubular epithelial cells contribute toward fibrosis by activating THBS1-CD47 signalling [23], while intermittent hypoxia has been shown to induce THBS1 expression and fibroblast activation, thereby inducing myocardial fibrosis in mice [24]. In this study, we analysed THBS1 expression in hypertrophic scars and normal tissues from 30 patients with hypertrophic scars. Compared to normal skin tissue and fibroblasts, THBS1 expression was dramatically upregulated in scar tissue and in hypertrophic scar fibroblasts; indeed, real-time PCR confirmed that THBS1 mRNA levels were almost 2.19 times higher in hypertrophic scar tissues than in normal tissues Therefore, we concluded that THBS1 may play a regulatory role in hypertrophic scarring.

Previous studies have demonstrated that THBS1 can stimulate the migration and proliferation of vascular endothelial cells and the formation of capillary buds *in vitro* [25]. In addition, THBS1 was found to promote the proliferation of RAW264.7 cells and inhibit their apoptosis [26], as well as promote the proliferation, migration, growth, and survival of tumour cells *in vivo* [27]. Here, we discovered that THBS1 overexpression can significantly induce the proliferation and migration of hypertrophic scar fibroblasts while inhibiting their apoptosis. In particular, the proliferation of fibroblasts increased by 28% 24 h after THBS1 transfection, while apoptosis decreased by 89% and migration increased by 62%. Conversely, THBS1 silencing inhibited the proliferation and migration of fibroblasts to a certain extent and upregulated apoptosis. These findings are consistent with a previous study which reported that THBS1 and TGF-*β*1 have similar effects on fibroblast migration [13].

THBS1 is also known to be involved in the regulation of multiple signalling pathways [28, 29] and antagonize VEGF in several important ways: inhibiting its release from the ECM, direct interaction, and inhibiting signal transduction [30–32]. The very low-density lipoprotein receptor inside the membrane of capillary endothelial cells binds TSP-1 and mediates the inhibition of cell cycle progression via the Akt/MAPK pathway [33]; therefore, targeting the TSP-1/CD47/NO signalling axis may promote the healing of skin grafts [30]. Since TGF-*β*1 plays a core role in fibrosis and THBS1 is thought to activate TGF-*β*1 in various fibrotic diseases [34], modulating THBS1 expression may represent an effective treatment option [35]. In this study, we confirmed that THBS1 regulates the growth and migration of hypertrophic scar fibroblasts and significantly promotes TGF-*β*1 expression. Furthermore, the downregulation of THBS1 expression *in vivo* was found to significantly inhibit the occurrence and development of bleomycin-induced hypertrophic scars in mice as well as TGF-*β*1 expression. Since the clinical sample size used in this study was relatively small, follow-up studies are required to confirm these findings and to explore the specific mechanisms via which THBS1 affects the growth and migration of hypertrophic scar fibroblasts *in vitro*. Moreover, the regulatory effects of THBS1 on collagen deposition, inflammation, and other important factors involved in hypertrophic scar formation merit further research.

## 5. Conclusions

In this study, we demonstrated that THBS1 expression is significantly upregulated in hypertrophic scars and can promote their development by inducing fibroblast growth and migration. Therefore, our findings suggest that THBS1 could be an ideal target for treating hypertrophic scarring.

## Data Availability

All data from this article can be supplied by the author upon reasonable request.
